# Verifying OpenJDK’s Sort Method for Generic Collections

**DOI:** 10.1007/s10817-017-9426-4

**Published:** 2017-08-31

**Authors:** Stijn de Gouw, Frank S. de Boer, Richard Bubel, Reiner Hähnle, Jurriaan Rot, Dominic Steinhöfel

**Affiliations:** 1 CWI, Science Park 123, 1098 XG Amsterdam, Netherlands; 2 Studiecentrum Utrecht, Open University, Vondellaan 202, 3521 GZ Utrecht, Netherlands; 3 Leiden University, Niels Bohrweg 1, 2333 CA Leiden, Netherlands; 4 Radboud University, Toernooiveld 212, 6525 EC Nijmegen, Netherlands; 5 Technische Universität Darmstadt, Hochschulstraße 10, 64289 Darmstadt, Germany

**Keywords:** Program verification, Specification, Case study, Theorem proving

## Abstract

TimSort is the main sorting algorithm provided by the Java standard library and many other programming frameworks. Our original goal was functional verification of TimSort with mechanical proofs. However, during our verification attempt we discovered a bug which causes the implementation to crash by an uncaught exception. In this paper, we identify conditions under which the bug occurs, and from this we derive a bug-free version that does not compromise performance. We formally specify the new version and verify termination and the absence of exceptions including the bug. This verification is carried out mechanically with KeY, a state-of-the-art interactive verification tool for Java. We provide a detailed description and analysis of the proofs. The complexity of the proofs required extensions and new capabilities in KeY, including symbolic state merging.

## Introduction

Among the arguments that are routinely invoked against the usage of formal software verification one can find the following: it is expensive, it is not worthwhile (compared to its cost), it is less effective than bug finding (e.g., by testing, static analysis, or model checking), it does not work for “real” software. In this article we present a case study in formal verification demonstrating that none of these arguments holds up in general and, on the contrary, formal specification and verification of real software is possible and can very well pay off.

We perform functional verification with mechanical proofs of TimSort, the sorting algorithm for generic collections in the Java standard library. Because of the complexity of the code under verification, it is essential to break down the problem into sub-tasks of manageable size. This is achieved with *contract-based deductive verification* [4], where the functionality and the side effects of each method are precisely specified with expressive first-order contracts. In addition, each class is equipped with an invariant that has to be re-established by each method upon termination. These formal specifications are expressed in the Java Modeling Language (JML) [14].

We use the state-of-art Java verification tool KeY [1], a semi-automatic, interactive theorem prover, which covers nearly full sequential Java. KeY typically finds more than 99% of the proof steps automatically (see Sect. 5), while the remaining ones are performed interactively by a human expert. This is facilitated by the use in KeY of symbolic execution plus first-order reasoning as its proof paradigm. It results in a close correspondence between proof nodes and symbolic program states which brings the experience of program verification somewhat close to the activity of debugging.

The work presented here was motivated by our recent success to verify executable Java versions of counting sort and radix sort in KeY with manageable effort [12]. As a further challenge, we planned to verify a complicated sorting algorithm taken from the widely used OpenJDK core library. It turns out that *the default implementation* of Java’s java.util.Arrays.sort() and java.util.Collection.sort() methods is an ideal candidate: it is based on a complex combination of merge sort and insertion sort [15, 19]. It had a bug history (see www.bugs.java.com/view_bug.do?bug_id=8011944), but was reported as fixed as of Java 8. We decided to verify the actual implementation with only two minor modifications: we stripped the code of generics and we modified one execution path that is irrelevant to the sorting result (see Sect. 7 for details). Otherwise, the verified Java code is identical to the library code and fully executable. The implementation is described in detail in Sect. 2.

During our verification attempt we discovered that the fix to the bug mentioned above is in fact not working [13] and that the “fixed” version crashes with an uncaught top-level exception on certain inputs. We succeeded to identify conditions under which the bug occurs (Sect. 3). From our analysis we could derive a bug-free version that does not compromise performance. The bug as reported in [13] led to different kinds of fixes in different languages, including Java, Android and Python. We review the reactions in Sect. 4.1, and then provide a detailed description of the proof that the fixed Java code terminates properly and does not raise any exception in Sect. 4.3. This includes two auxiliary methods that could not be proven correct in [13]. The Android community provided an alternative fix, which we proved to be correct as well, as reported in Sect. 4.4.

We provide a detailed account of the proof statistics in Sect. 5. They show that the symbolic state merging technique recently implemented in KeY [22] can successfully mitigate state explosion during symbolic execution. In addition, we analyze the nature of user interactions, thereby providing indicators where prospects for automation lie and how the individual style of proof engineers can influence efficiency of the proof effort.

In Sect. 6 we draw lessons from our experience of proving TimSort. These concern the development of formal specifications, the choice of integer semantics and how to deal with state explosion. While our case study shows that formal specification and verification of real Java library code is possible and pays off, it also highlighted a number of limitations to current verification technology. These are discussed in Sect. 7.

In addition to fixing the bug, our verification effort exhibited further potential issues with TimSort. We point out some recommendations to the maintainers of TimSort in Sect. 8. Related work is discussed in Sect. 9 and in Sect. 10 we draw conclusions.

This paper is a revised and extended version of [13]. The extension includes a more detailed description and (statistical) analysis of the proofs, including a comparison between the old proof and several new proofs based on branch merging techniques; a (mechanic) proof for all methods, including mergeLo and mergeHi, which only became possible after several new techniques (branch merging, a new do-while rule) were added to KeY; and a mechanic proof of the Android version.

## Implementation of TimSort

The default implementation of java.util.Arrays.sort for non-primitive types is TimSort, a hybrid sorting algorithm based on merge sort and insertion sort. The algorithm sorts a specified segment of the input array incrementally from left to right based on consecutive (disjoint) *runs*: segments of the array that are already sorted. If these runs are not large enough, they are extended using binary insertion sort. The starting positions and the lengths of the generated runs are stored on a stack. During execution some of these runs are merged, triggered by a condition on the top elements of the stack. In the end, all runs are merged, yielding a sorted array.

Now we explain the algorithm in detail, focusing on the central parts of the Java implementation. The interface of TimSort is given by the two static methods on lines 1 and 29 of Listing 1. The main method of TimSort is shown in Listing 1 (with original comments), where a is the input array. The parameters lo and hi are the lower bound (inclusive) and upper bound (exclusive) of the part of a that must be sorted. To sort the entire array, they can be omitted, see lines 29–31.

The stack of runs is encapsulated by the object variable ts which is constructed in line 5. It is represented by two instance variables runBase and runLen of the object ts, that refer to arrays of integers. These arrays runBase and runLen contain respectively the starting positions and the lengths of the runs on the stack.^1^ The (variable) size of the stack is kept in the instance variable stackSize. The allocated length of these arrays runBase.length and runLen.length is determined in the constructor of ts based on the length of the input array. 
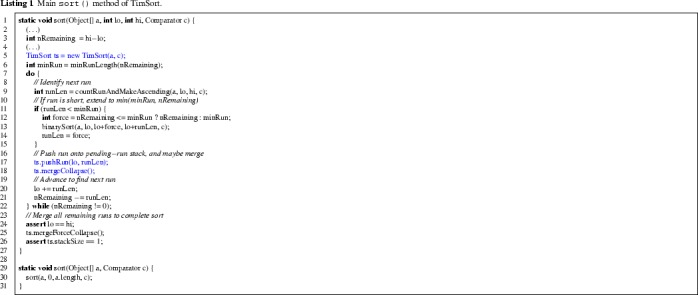


In each loop iteration, the next run is constructed. In line 9 a maximal run from the current position lo is constructed by identifying a maximal descending or ascending segment, reversing the order in case of a descending one. If the resulting run is too short (that is, less than minRun) then it is extended to a run of length minRun using binary insertion sort (“binary” refers to the fact that it uses binary search). The variable nRemaining denotes the number of elements yet to be processed. In line 17 the starting position and the length of the run is pushed onto the stack of the object variable ts by method pushRun in Listing 2.
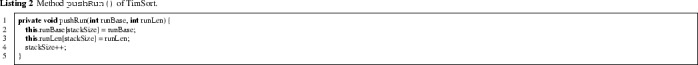


In line 18, the method mergeCollapse is called, which repeatedly merges runs until the top three elements of the stack satisfy the conditions given in lines 4 and 5 of Listing 4, for i = stackSize. Actual merging is done by a call to mergeAt(n), which merges the two runs whose lengths are represented respectively by runLen[n] and runLen[n+1]. Without going into detail, we note that mergeAt (see Listing 3) features an optimisation that identifies parts of the two runs that do not need to be considered for merging, and then merges either from left to right or from right to left (by a call to mergeLo or mergeHi respectively), using a temporary array holding a copy of the smaller of the two.
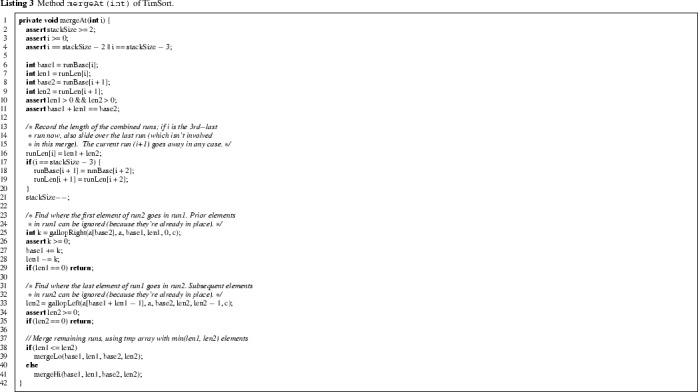


We describe the merging pattern of mergeCollapse. To simplify the notation, let $\texttt {runLen[n-1]}=C$, $\texttt {runLen[n]}=D$, and $\texttt {runLen[n+1]}=E$, representing the lengths of the top three runs on the stack (observe that n = stackSize-2 at the beginning of each iteration). The condition to be established is that $C > D + E$ and $D > E$. The loop achieves this by distinguishing the following cases:

1. If $C\le D+E$ and $C<E$ then the runs at n-1 and n are merged.
2. If $C\le D+E$ and $C\ge E$ then the runs at n and n+1 are merged.
3. If $C> D+E$ and $D\le E$ then the runs at n and n+1 are merged.
4. If $C>D+E$ and $D>E$ then the loop exits.

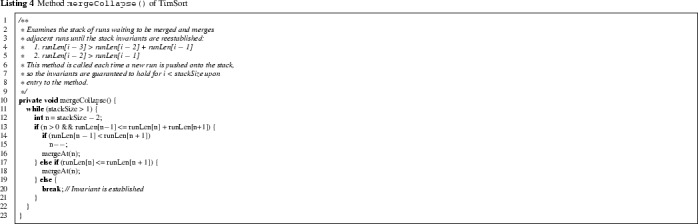


After exiting the main loop in Listing 1, the entire input array has been processed, but there may still be pending runs on the stack. These runs are finally merged by the call to mergeForceCollapse (Line 25 of Listing 1), which repeatedly merges runs on the stack (either the top two or the two below it) until only a single run remains, representing the sorted array.

## Breaking the Invariant

As explained in the previous section, the method mergeCollapse establishes the following postcondition:

1. $$\texttt {runLen[i - 3] > runLen[i - 2] + runLen[i - 1]/}$$
2. $$\texttt {runLen[i - 2] > runLen[i - 1]}$$

for $\texttt {i = stackSize}$. However, as the comments in lines 1–9 of Listing 4 suggest, the intention is that the above conditions are “re-established” by mergeCollapse for *all*$\texttt {i <= stackSize}$. More precisely, the above conditions for $\texttt {i <= stackSize}$ should be a *loop invariant* of the main loop of the sort method (Listing 1). After the call to pushRun in the sort method (line 17) the loop invariant is temporarily broken (for the case i = stackSize), and the intention is that it is restored by the call to mergeCollapse (line 18).

The above loop invariant is fundamental to TimSort, hence we sometimes refer to it simply as *the invariant*. We shall also refer to specific elements of runLen satisfying the *(element) invariant*, meaning that such an element is greater than the next one and greater than the sum of the next two.

The invariant is used to compute the length of runLen, as explained in more detail in Sect. 3.1. However, the method mergeCollapse does not re-establish the invariant in general, contrary to what is suggested in the comments. To see this, consider the situation where runLen consists of

$$\begin{aligned} 120,\, 80,\, 25,\, 20,\, 30 \end{aligned}$$

on entry of mergeCollapse, directly after the entry 30 was added by pushRun. In the first iteration of the mergeCollapse loop there will be a merge at entry 25, since $25 \le 20 + 30$ and $25 < 30$, resulting in (Listing 4, conditions in lines 13 and 14 are evaluated to true causing lines 15 and 16 to be executed):

$$\begin{aligned} 120^\times ,\, 80,\, 45,\, 30. \end{aligned}$$

We annotate an entry with the superscript $\times$ if it does not satisfy the element invariant. In the second iteration the element invariant is satisfied at entries 80 and 45 (i.e., conditions at lines 13 and 17 are evaluated to false and line 20 is executed), because $80 > 45 + 30$ and $45 > 30$, so mergeCollapse terminates. The element invariant does not hold at entry 120, however, since $120 \le 80 + 45$. Thus, mergeCollapse has not fully restored the loop invariant of sort.

More generally, an error (a violation of the invariant) can only be introduced by merging the third-to-last and second-to-last element and requires precisely four elements after the position of the error, i.e., at runLen[stackSize-5]. Indeed, suppose runLen consists of four elements *A*, *B*, *C*, *D* satisfying the invariant. We add a fifth element *E* to runLen using pushRun, after which mergeCollapse is called. The only possible situation in which an error can be introduced, is when $C \le D + E$ and $C <E$. In this case, *C* and *D* will be merged, yielding the stack

$$\begin{aligned} A,\,B,\,C+D,\,E. \end{aligned}$$

Then mergeCollapse checks whether the invariant is satisfied by the new three top elements. But *A* is not among those, so it is not checked whether $A > B + C + D$. As shown by the above example, this latter inequality need not hold in general.

### The Length of runLen

The invariant affects the maximal size of the stack of run lengths during execution; recall that this stack is implemented by runLen and stackSize. The length of runLen is declared in the constructor of TimSort, based on the length of the input array a and, as shown below, on the assumption that the invariant holds. For performance reasons it is crucial to choose runLen.length as small as possible (but so that stackSize never exceeds it). The original Java implementation is shown in Listing 5 (the number 24 replaced the previous value of 19 in a later update, see Sect. 3.2):
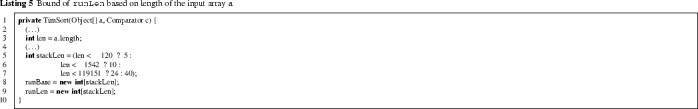


We explain the bounds, assuming the invariant to hold. Consider the sequence $(b_i)_{i \ge 0}$, defined inductively by $b_0 = 0$, $b_1 = 16$ and $b_{i+2} = b_{i+1} + b_i + 1$. The number 16 is a general lower bound on the run lengths $b_i$, and $b_0, \ldots , b_n$ are lower bounds on the run lengths in an array runLen of length *n* that satisfy the invariant; more precisely, $b_{i-1} \le \texttt {runLen[n-i]}$ for all *i* with $0 < i \le n$.

Let runLen be a run length array arising during execution, assume it satisfies the invariant, and let $n = \texttt {stackSize}$. We claim that for any number *B* such that $1 + \sum _{i=0}^B b_i > \texttt {a.length}$ we have $n \le B$ throughout execution. This means that *B* is a safe bound, since the number of stack entries never exceeds *B*.

The crucial property of the sequence $$(b_i)$$ is that throughout the whole execution we have $$\sum _{i=0}^{n-1} b_i < \sum _{i=0}^{n-1} \texttt {runLen[i]}$$ using that $$b_0 = 0 < $$runLen[n-1] and $$b_{i-1} \le \texttt {runLen[n-i]}$$. Moreover, we have $$\sum _{i = 0}^{n-1} \texttt {runLen[i]} \le \texttt {a.length}$$ since the runs in runLen are disjoint segments of a. Now for any *B* chosen as above, we have $$\sum _{i =0}^{n-1} b_i< \sum _{i=0}^{n-1} \texttt {runLen[i]} \le \texttt {a.length} < 1+ \sum _{i=0}^B b_i$$ and thus $$n \le B$$. Hence, we can safely take $$\texttt {runLen.length}$$ to be the least *B* such that $$1 + \sum _{i=0}^B b_i > \texttt {a.length}$$. If $$\texttt {a.length} < 120$$ we thus have 4 as the minimal choice of the bound, for $$\texttt {a.length} < 1542$$ it is 9, etc. This shows that the bounds used in OpenJDK (Listing 5) are slightly sub-optimal (off by 1). The default value 40 (39 is safe) is based on the maximum $$2^{31}-1$$ of integers in Java.

### Breaking TimSort

We saw that the invariant is not restored by mergeCollapse, contrary to what is stated in the comments, while the bound of runLen in the constructor of TimSort is based on the assumption that the invariant holds. It is possible to exploit the fact that the invariant breaks by construction of a “bad” case [13]. In fact, as sketched in [13], this actually gives rise to the worst case, meaning that for a given input length the construction yields an input array that reaches the largest possible stackSize during execution.

We implemented this construction of worst case inputs. The required stack sizes for the worst case in terms of the input length are shown in Table 1. Note in particular that these inputs require a greater bound on runLen than is obtained by the analysis in the previous subsection (which assumed the invariant to hold). The third row shows the declared bounds in the TimSort implementation (see Listing 5). The bound 19 was updated to 24 after a bug report.^2^

**Table 1:** Required stack size versus declared bounds on runLen

Generated array size	64	128	160	65536	131072	67108864	1073741824
Required stack size	3	4	5	21	23	41	49
runLen.length	5	10	10	19 (24)	40	40	40

For instance, the worst case of length 160 requires a stack size of 5, and thus the declared runLen.length = 10 suffices. Further observe that 19 does not suffice for the worst case for arrays of length 65536, whereas 24 does.^3^ For the worst case of length 67108864, the declared bound 40 does not suffice, and running TimSort yields the unpleasant result shown in Listing 6.
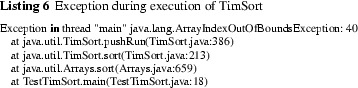


## Verification of a Fixed Version

We discuss possible approaches to fix TimSort and describe how one of these fixes was formally verified. To be specific, we proved mechanically that TimSort terminates normally for any input, i.e., no ArrayIndexOutOfBoundsException is thrown, see Listing 7. We have not proven that TimSort ensures the sortedness and permutation properties. However, as will become clear in Sect. 4.3 below, even the proof of absence of top-level exceptions required verification of a considerable amount of non-trivial functional specifications (500+ lines, see Table 2). The reason is that ensuring that the exception is never thrown depends on a complex wellformedness property that must be maintained. Hence, we do not think this specification and verification effort could have been achieved with “lightweight” verification tools [6].
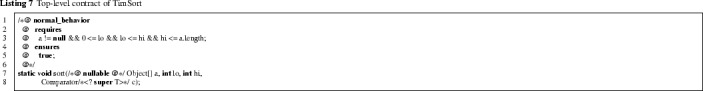


### Different Ways to Fix TimSort

In [13] we suggested two possible fixes of the TimSort bug. The first was to use larger stack sizes based on an operational pen-and-paper worst case analysis of the implementation. While the code change is trivial (modification of a few integer numbers in the allocation table), we did not favor this solution as it fixes the symptom, but not the underlying problem that the fundamental invariant of TimSort is broken. In short, when adopting this fix (as done in the Java OpenJDK), one uses an algorithm that by itself is not fully understood why and how it works. We do not know how to formulate a correct invariant for the original implementation of the algorithm.

We favored the second suggestion which is to formalize the invariant as originally intended and to fix the code of the method mergeCollapse that is responsible for re-establishing the invariant. We were able to formally and mechanically prove that this fixed version of the algorithm is correct in the sense that the stack lengths are sufficient and no ArrayIndexOutOfBoundsException is thrown. We describe this fix and its verification in Sect. 4.3 below.

In the aftermath of our discovery, it turned out that the bug was present in several implementations of TimSort. Besides in (Open)JDK,^4^ the bug was present in (1) its original Python implementation,^5^ (2) Android,^6^ (3) an independent Java implementation used by Apache Lucene,^7^ as well as (4) a Haskell implementation.^8^

All of these projects fixed the bug within a short time frame. The OpenJDK project was the only one where the bug was fixed by just increasing the allocated array lengths, which is in our opinion sub-optimal, and there is no machine checked proof of that fix. All other projects implemented our second suggestion and fixed the underlying problem. Notably, the Android fix varies from our proposal, but we were able to mechanically verify their fix with only minor modifications to the specifications and proofs of our fix. We discuss the Android fix in detail in Sect. 4.4. We do not know the reason for the alternative fix as the comment discussing their solution refers to an internal Google issue tracker.

### Verification in KeY

Before we start to explain the verification of TimSort in detail, we briefly sketch the verification process in KeY. In particular, we clarify the notion of proof obligation and explain its generic form. We start by explaining that KeY is based on symbolic execution rather than on verification condition generation. For each method a formula in Java Dynamic Logic (JavaDL) is generated, which is valid if and only if the implementation adheres to its specification. The formula is then given to KeY ’s theorem prover to be proven valid. The formula contains the source code of the method to be verified as first class citizen and the calculus rules concerned with program elimination implement a symbolic interpreter, i.e., the whole program elimination is an integral part of the logic calculus itself and not an external entity. The advantage of integrating symbolic execution and first-order reasoning into a logic calculus is that first-order reasoning and symbolic execution rules can be seamlessly interleaved. This allows KeY to simplify intermediate states eagerly and to close infeasible paths early.

The simplified form of a proof obligation for a method *m* is

$$\begin{aligned} ( pre \wedge inv ) \rightarrow \langle m( args ); \rangle ( post \wedge inv ) \end{aligned}$$

with $pre , post$ and $inv$ being the method precondition, postcondition and the class invariant, respectively. In JML, the specification language used by KeY, a precondition is given by a requires clause, and a post-condition is given by ensures. To avoid manually adding the class invariant at all these points, JML offers an invariant keyword which *implicitly* conjoins the class invariant to all pre- and post-conditions.

The formula expresses that, if a method *m* is invoked in a state where the precondition and invariant hold, then *m* terminates normally (not throwing an exception) and in its final state the postcondition and the class invariant holds. This dynamic logic formula is equivalent to the Hoare triple

$$\begin{aligned} \{ pre \wedge inv \}~m( args )~\{ post \wedge inv \} \end{aligned}$$

plus termination.

Such formulas had to be proven valid for all methods of TimSort. Roughly, during verification the method body of *m* is symbolically executed to translate the above formula into a pure first order formula. When symbolic execution requires to execute a method call statement, the contract of the invoked method was used instead of inlining its implementation. Using a contract involves to show that at the point the precondition of the called method as well as the invariant of the receiver object holds. Symbolic execution then continues with the next statement after the method invocation, assuming that the method’s postcondition and the class invariant holds.

**Fig. 1:**
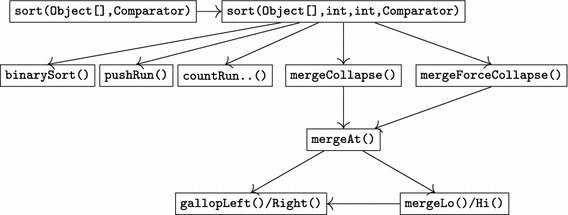
Call dependencies of TimSort methods (excluding methods: minRunLength, rangeCheck and invocation of Java library methods like System.arraycopy)

We conclude this section with a brief overview of the required proof-obligations for verification of TimSort, and their interplay. Figure 1 provides a (simplified) call graph. For each of the methods we have to prove that they adhere to their specification and in particular preserve the invariant. The methods directly relevant for the bug are the methods pushRun (where the exception was thrown) and mergeCollapse, which failed in its original implementation to re-establish the invariant. The specification and verification of these methods is explained in detail in Sects. 4.3 and 4.4.

The methods mergeLo/Hi do not change the runLen array or other program locations occurring in the invariant at all and thus cannot invalidate the invariant. Their verification proved surprisingly challenging because of their complex control flow which caused the number of symbolic paths to explode. In order to be able to prove these methods, extensions to the KeY verification system were necessary, namely, an improved rule to verify do-while loops (see Sect. 5.1) and state-merging (see Sect. 6.3).

The verification of these methods was mostly necessary to exclude the presence of implicit run-time exceptions and only a few of the listed methods mentioned in the previous two paragraphs modify fields occurring in the invariant. Their method contracts serve mostly to ensure that no NullPointerExceptions are thrown and that accesses to the array to be sorted are within bounds.

### Verification of the Code that Re-establishes the Invariant

In Sect. 3 we showed that mergeCollapse does not fully re-establish the invariant, which led to an ArrayIndexOutOfBoundsException in pushRun. Now we fix mergeCollapse so that the invariant of the main sorting loop (Listing 1) *is* re-established, formally specify the new implementation in JML and provide a correctness proof, focusing on the most important specifications and proof obligations. (In the specification listings shown below we omitted some irrelevant lines; the specifications are otherwise unaltered). This formal proof has been fully mechanized in the theorem prover KeY [1]. Statistics of the verification effort and insights drawn from it are discussed in Sects. 5 and 6.
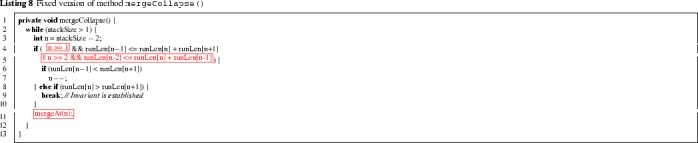


Listing 8 shows the fixed version of mergeCollapse. The main idea is to check validity of the element invariant on the top *four* elements of runLen (lines 4–5 and 8), instead of only the top three, as in the original implementation. The question arises: why is checking the last four runs sufficient? Initially, the precondition of mergeCollapse guarantees that all but the last three runs satisfy the element invariant. After mergeAt, the entry of runLen at index stackSize-2 or stackSize-1 may be modified, but runs at earlier indices remain intact. Thus the element invariant of runLen[stackSize-4] might not hold after merging, but the element invariant of earlier runs is not affected by the merging. This is the basis for checking the element invariant on the last four runs. Merging continues until the top four elements satisfy the invariant, at which point we break out of the merging loop (line 9). We prove below that this ensures that *all* runs satisfy the invariant.

To obtain a human readable specification and a feasible (mechanized) proof, we introduce suitable abstractions using the following auxiliary predicates:

**Table:** 

Predicate name	Predicate definition
$\text{ elemBiggerThanNextTwo }(arr, idx)$	$(0 \le idx \wedge idx+2 < arr.length) \rightarrow$
	$$arr[idx] > arr[idx+1] + arr[idx+2]$$
$\text{ elemBiggerThanNext }(arr, idx)$	$0 \le idx \wedge idx+1 < arr.length \rightarrow$
	$$arr[idx] > arr[idx+1]$$
$\text{ elemLargerThanBound }(arr, idx, v)$	$0 \le idx < arr.length \rightarrow arr[idx] \ge v$
$\text{ elemInv }(arr, idx, v)$	$\text{ elemBiggerThanNextTwo }(arr, idx) \wedge$
	$$\text{ elemBiggerThanNext }(arr, idx) \wedge $$
	$$\text{ elemLargerThanBound }(arr, idx, v)$$

The predicate $\text{ elemInv }(\texttt {+}runLen+,\mathtt {i}, 16)$ holds when runLen[i] satisfies the element invariant as defined in Sect. 3, and has length at least 16 (the lower bound on the minimal run length). Aided by these predicates we are ready to express the formal specification, beginning with the main sorting loop, which contains the fundamental invariant of TimSort.

*Invariant of Main Sorting Loop* We now specify the main sorting loop (Listing 1), formalising the invariant discussed in Sect. 3. Listing 9 shows the loop invariant in JML (note the use of the JML keyword loop_invariant). The crucial lines 6–10 express that all elements in runLen satisfy the invariant.

The local variable lo points to the index in the input array a of the first element that has yet to be processed, thus a has been partitioned into runs from index^9^old(lo) to lo. Since JML by default uses Java integer types, which can overflow, we need to make sure this does not happen by casting those expressions that potentially can overflow to $\backslash \texttt {bigint}$ (the $\backslash \texttt {bigint}$ type represent mathematical integers). Furthermore nRemaining counts the number of elements still to be processed. This explains line 4 and 5. Line 11–12 specify that if there are remaining elements, all runs in runLen have a length of at least 16.
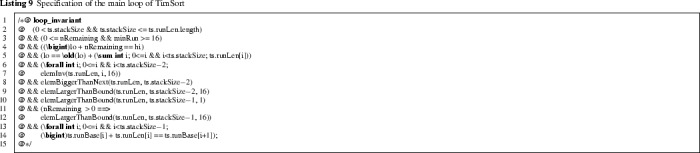
 The class invariant is formalised next. It is roughly a weaker version of this loop invariant.

*Class Invariant* As mentioned above, a class invariant is a property that all instances of a class should satisfy and which must be preserved by each instance method, i.e., if it holds before a method invocation then it must also hold after termination of the method.^10^ This means the class invariant is implicitly contained in a method’s pre- and postcondition.

A seemingly natural candidate for the class invariant states that *all* runs on the stack satisfy the element invariant and have a length of at least 16, like lines 6–10 of the sort(..) loop invariant. The method pushRun critically relies on this invariant, to ensure that runLen is sufficiently long to push a new run on the stack (otherwise, it throws the ArrayIndexOutOfBoundsException). However, this class invariant is not preserved by pushRun. Further, inside the loop of mergeCollapse (Listing 8) the mergeAt method is called, so the class invariant must hold after it. But the merge could result in a new entry at the one-but-last index stackSize-1 in runLen, thus the element invariant for the last four runs might be broken after mergeAt. Finally, the last run pushed on the stack in the main sorting loop (Listing 1) can be shorter than 16 if fewer items remain. The class invariant given in Listing 10 addresses all this.
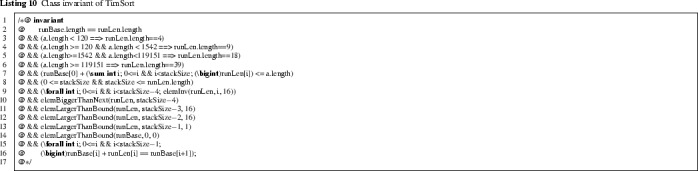


Lines 3–6 specify the length of runLen in terms of the length of the input array a. Line 8 formalize the property that the length of all runs together (the sum of all run lengths) does not exceed a.length. Line 8 contains bounds for stackSize. Line 9 expresses that all but the last four elements satisfy the element invariant. The properties satisfied by the last four elements are specified on lines 10–13. Lines 14–16 say that run $\mathtt {i}$ starts at runBase[i] and extends for runLen[i] elements.

*The* pushRun *method.*

This method adds a new run of length runLen to the stack starting at index runBase.^11^ Lines 4–5 of Listing 11 express that the starting index of the new run (runBase) directly follows after the end index of the last run (at index stackSize-1 in this.runLen and this.runBase). The assignable clause indicates which locations can be modified; it entails that previous runs on the stack are unchanged.
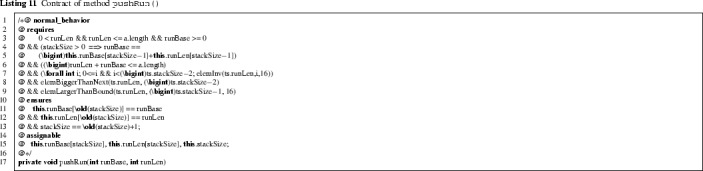


*The*mergeCollapse*method.* The new implementation of mergeCollapse restores the invariant at all elements in runLen; this is stated in lines 6–7 of Listing 12. Since the method mergeCollapse only merges existing runs, the sum of all run lengths should be preserved (lines 8–9). Line 10 expresses that the length of the last run on the stack after merging never decreases (merging increases it). This is needed to ensure that all runs, except possibly the very last one, have length $\ge 16$.
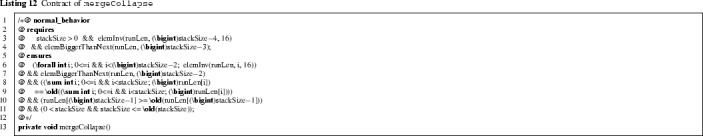


The loop invariant of mergeCollapse is given in Listing 13. As discussed above, merging preserves the sum of all run lengths (lines 2–3). Line 4 expresses that all but the last four runs satisfy the element invariant: a merge at index $\texttt {stackSize-3}$ (*before* merging) can break the invariant of the run at index stackSize-4*after* merging (beware: stackSize was decreased). Lines 5–8 state the conditions satisfied by the last four runs. Lines 9–10 specify consistency between runLen and runBase. Line 11 states that stackSize can only decrease through merging.
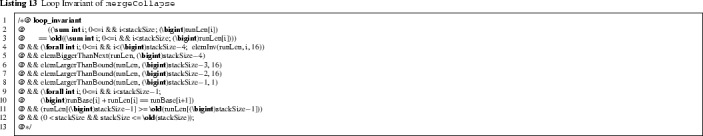


In order to prove the contract of mergeCollapse we make use of the contract of mergeAt in Listing 14. The postcondition formalizes the first three cases of the merging pattern of mergeCollapse as described in Sect. 2. It allows us to formally prove that, by repeated application of this merging pattern, the loop invariant of mergeCollapse in Listing 13 is established again.
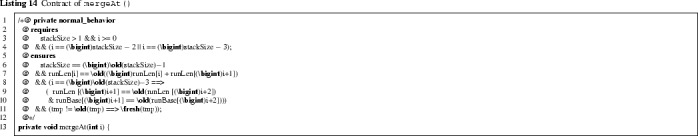


To prove that each method satisfies its contract, several verification conditions must be established. We discuss the two most important ones. The first states that on entry of pushRun, the stackSize must be smaller than the stack length: 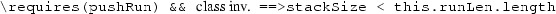
This is a crucial property: the ArrayIndexOutOfBoundsException of Listing 6 was caused by its violation.

#### Proof

Line 8 of the class invariant implies $\texttt {stackSize} \le \texttt {this.runLen.length}$. We derive a contradiction from $\texttt {stackSize} = \texttt {this.runLen.length}$ by considering four cases: $\texttt {a.length} < 120$, or $\texttt {a.length} \ge 120\texttt { \& \& }{} \texttt {a.length} < 1542$, or $\texttt {a.length} \ge 1542\texttt { \& \& }{} \texttt {a.length} < 119151$, or $\texttt {a.length} \ge 119151$. We detail the case $\texttt {a.length} < 120$, the other cases are analogous. Since $\texttt {a.length} < 120$, line 3 of the class invariant implies $\texttt {stackSize} = \texttt {this.runLen.length} = 4$.

Let $${\texttt {SUM} = \texttt {this.runLen[0]}} \ldots + \texttt {this.runLen[3]}$$. Suitable instances of lines 15–16 of the class invariant imply $$\texttt {this.runBase[3]} + \texttt {this.runLen[3]}$$$$= \texttt {this.runBase[0]} + \texttt {SUM}$$. Together with line 14 of the class invariant and lines 4–5 of the pushRun contract we get $$\texttt {runLen} + \texttt {SUM} < 120$$. But the $$\backslash \texttt {requires}$$ clause of pushRun implies $$\texttt {runLen} > 0$$, so $$\texttt {SUM} < 119$$. The $$\backslash \texttt {requires}$$ clause also implies $$\texttt {runLen[3]} \ge 16$$ (line 9), $$\texttt {runLen[2]} \ge 17$$ (line 8), $$\texttt {runLen[1]} \ge 34$$ and $$\texttt {runLen[0]} \ge 52$$ (line 7). So $$\texttt {SUM} \ge 16+17+34+52 = 119$$, a contradiction. $$\square $$

The second verification condition arises from the break statement in the loop of the method mergeCollapse (Listing 8, line 9). At that point the guards on lines 4–5 are false, the one on line 8 is true, and the $\backslash \texttt {ensures}$ clause of mergeCollapse (which implies that the invariant holds for all runs in runLen) must be proven: 
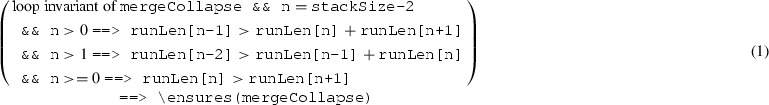


#### Proof

Preservation of sums (lines 8–9 of $\backslash \texttt {ensures}$) follows directly from lines 2–3 of the loop invariant. Lines 10–11 of $\backslash \texttt {ensures}$ are implied by lines 11–12 of the loop invariant. The property elemBiggerThanNext(runLen,stackSize-2) follows directly from $\texttt {n}>= 0 \texttt { ==> runLen[n]} > \texttt {runLen[n+1]}$. We show by cases that 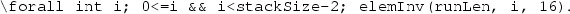

- $\texttt {i} < \texttt {stackSize-4}$: from line 4 of the loop invariant.
- $\texttt {i} = \texttt {stackSize-4}$: from line 3 of the premise. The original mergeCollapse implementation (Listing 4) did not cover this case, which was the root cause that the invariant $\texttt {elemInv(runLen, i, 16)}$ could be false for some i.
- $\texttt {i} = \texttt {stackSize-3}$: from the line 4 of the premise.$\square$

*Preservation of the Main Loop Invariant* The final proof obligation we discuss states that the loop invariant (Listing 9) of the main sorting loop (Listing 1) is preserved by the loop body. Line 2 follows from line 11 of the contract of mergeCollapse (Listing 12) and line 8 of the class invariant (Listing 10). Line 3 follows from the contract of minRunLength and and line 9–14 of the main loop. Line 4 follows from line 20, 21 of the main loop. For line 5, notice that pushRun increases the sum of run lengths with (the local variable passed as parameter) runLen (see line 12–15 of its contract, Listing 11); this sum is preserved by mergeCollapse (line 8 and 9 of its contract); finally in line 20 of the main loop, lo is incremented with the value of runLen.

Line 6–7, formalising the crucial part of the invariant, follow from line 6 of the contract of mergeCollapse. Line 8 follows from line 7 of the contract of mergeCollapse. Lines 12–13 follow from lines 12–13 of the class invariant. Lines 11-12 follow from lines 11–15 and 21 of the main loop. Finally, lines 13–14 follows from lines 15–16 of the class invariant.

Of course, all proof obligations described above (plus all others) were formally shown in KeY.

### The Android Fix

In the Android implementation of TimSort, the bug was corrected by adapting the method mergeCollapse in a different way than proposed in Listing 8. We discuss this new version and its correctness, which we have proved in KeY.
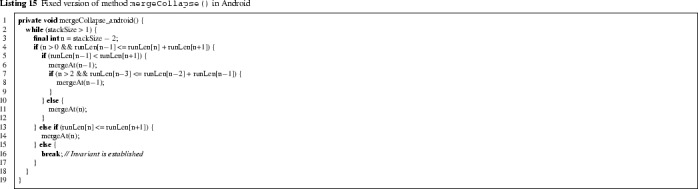


The above method also checks that the last four elements of runLen satisfy the invariant, but at a different time than the corrected version in Listing 8. Suppose that on entry of the loop, the last five elements of runLen are *A*, *B*, *C*, *D*, *E*. As explained in Sect. 3, the invariant can be broken at (the run of length) *A* by merging the runs *C* and *D*, which would happen in line 6. But in this case, the above method immediately checks whether the invariant holds at *A* (i.e., whether $A> B + C + D$), and if not, it merges the last two runs, yielding the run length stack $A,B,C+D+E$. Hence, the appropriate invariant for mergeCollapse_android ensures that the invariant holds at all but the last *three* elements of runLen. Indeed, it is obtained from the invariant of mergeCollapse in Listing 13 by replacing lines 4–5 by the following: 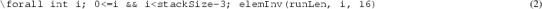


This is the only required change: the contract of mergeCollapse_android is the same as the contract of mergeCollapse. Hence, similar to the explanation of the correctness of mergeCollapse, the main verification condition arises from the break statement at line 16. At that point, the guards at line 4 and 13 are false, hence we must prove the following: 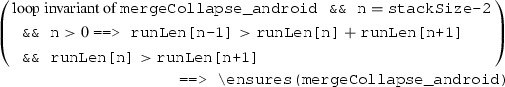
The invariant of mergeCollapse_android implies that of mergeCollapse, and, because of (2), it implies the left-hand side of the verification condition (1) discussed above in the correctness of mergeCollapse. Finally, since we have that 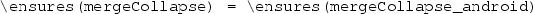 (their contracts are identical), the above verification condition follows from (1).

## Proof Statistics

Our analysis resulted in one of the largest case studies carried out so far in KeY, with nearly 3 million proof steps in total.^12^ The KeY proof targets the actual implementation in the OpenJDK standard library, rather than an idealized model of it. That implementation uses low-level bit-wise operations, abrupt termination of loops and arithmetic overflows. Our proof effort motivated several improvements to KeY, such as improved support for reasoning about operations on bit-vectors. Table 2 shows some general statistics about the proof.

Before discussing the proof statistics in detail, we briefly explain the automated proof search in KeY. KeY provides a semi-automated theorem prover that allows the user to work on the same proof representation as the automated part of the prover. This means that, in case the automation fails, the user can inspect the proof situation and apply some rules interactively to steer the proof search in the right direction and then restart the proof search strategies. The default strategy is cost based and implements a number of reasoning techniques with support for theories like integers (linear and to some part non-linear arithmetics) or finite sequences. The user can choose to run the strategies as-is or to use proof macros which constrain the strategies such that, in case of failed proof attempts, the result can be easier understood by a human. KeY also allows to invoke SMT solvers. In that case, a successful invocation closes the corresponding proof goal by referring to the SMT prover, but KeY does not check whether the obtained result is correct.

One reason for the large number of proof steps is their fine granularity. However, notice that only a relatively small number of steps was applied manually (column “Interact”). Most of the manual interactions are applications of elementary weakening rules (hiding large irrelevant formulas) to guide the automated proof search. Application of the majority of the elementary weakening rules could be automated by extending the current strategies to more aggressively detect subsumption and in particular, by allowing the strategies to eliminate equations of the kind $c \doteq t$ where *c* is a constant symbol not occurring anywhere else in the sequent.

Approximately 5–10% of the interactive proof steps required some ingenuity, such as introducing lemmas and finding suitable quantifier instantiations (column “Q-inst”). The columns “Call” and “Loop” show the number of rule applications concerning calls and loops encountered in symbolic execution paths (in total, i.e. both interactive and non-interactive applications count). Since multiple paths can lead to the same call, this is higher than the number of calls in the source code. The last two columns show the number of lines of specification and code (without comments).

In the proofs of some of the methods, we employed a novel symbolic state merging technique [22] for mitigating the path explosion problem during the symbolic execution (column “Merges”). In the remainder of this section, we describe the effect of state merging on the proof size (Sect. 5.1), and discuss the required user interactions in greater detail (Sect. 5.2).

**Table 2:** Lines of code and specification and proof statistics for TimSort methods

Method	Rule apps	Interact	Call	Loop	Q-inst	Merges	Spec	LoC
mergeLo	1,455,918	20,204	11	3	713	12	66	88
mergeHi	460,409	3312	13	3	478	5	62	92
mergeCollapse	348,774	1849	4	1	409	4	48	13
sort(a,lo,hi,c)	152,752	359	10	1	41	1	42	52
binarySort	92,593	323	2	2	10	0	27	35
mergeAt	63,309	794	4	0	155	6	32	39
pushRun	42,142	129	0	0	72	0	18	5
mergeForceColl	58,567	475	3	1	27	2	39	10
Other (sum)	292,058	1346	67	18	147	13	174	171
Total	2,966,522	28,791	114	29	2052	43	508	505

**Rule Apps**: Total number of rule applications

**Interact**: Number of interactively (manually) applied rules

**Call**/**Loop**/**Q-inst**/**Merges**: Numbers of encountered method calls/loop invariants/quantifier instantiations/merge rule applications (interactive *and* automatic rule apps. count).

**Spec** and **LoC:** Lines of Specification / Code

### Proof Size Reduction by State Merging

One of the main bottlenecks of symbolic execution is the path explosion problem [9]. It stems from the fact that symbolic execution must explore all symbolic paths of a program to achieve high coverage (in testing), respectively, soundness (in verification). The number of paths from the root to the leaves in a symbolic execution tree is usually exponential in the number of static branches of the executed program. By merging suitable proof nodes arising during symbolic execution, this problem can be mitigated (in Sect. 6.3, we describe the state merging framework implemented in KeY in some more detail.)

**Fig. 2:**
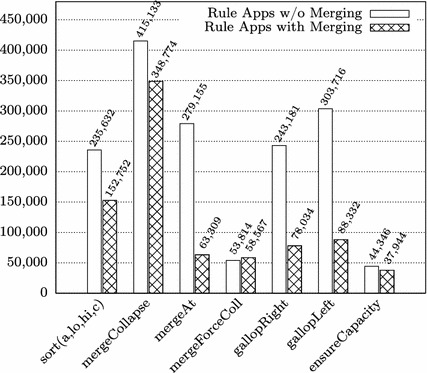
Comparison of proof sizes with and without state merging. Only proofs where state merging has been applied are considered

In [13], we used symbolic execution *without* state merging for proving the absence of exceptions in TimSort. For the present paper, we redid all the proofs in a version of KeY that features state merging techniques. Figure 2 shows a comparison of the proof sizes with and without state merging for all methods where merging has been applied. On average, we were able to reduce the proof sizes by 39%, with a maximum reduction of almost 80% in the case of mergeAt.

In most cases, we employed a state merging technique requiring very little human interaction and preserving full precision. The technique essentially combines differing values in the merged states to a value summary (so-called “if-then-else merging”). In the value summary, all differing values for a variable are remembered precisely, and are constrained by the path conditions of the nodes in the execution tree they originated from. For two methods, mergeForceCollapse and ensureCapacity, the number of proof nodes *increased* when applying merge techniques. This can be explained by the relatively small size of those proofs; the saved redundancy is insufficient for compensating the increased complexity in the merged nodes. By using a predicate abstraction merging technique, where differing values are abstracted from by user-supplied unary predicates, we managed to decrease the proof size by 15% through the choice of abstraction predicates tailored to the specific problem in ensureCapacity.

**Table 3:** Interactive versus total number of rule applications (per method)

Method	Rule apps	Interactive rule apps	Interactive rule apps (%)
mergeLo	1,455,918	20,204	1.39
mergeHi	460,409	3312	0.72
newMergeCollapse	348,774	1849	0.53
sort(a,lo,hi,c)	152,752	359	0.24
binarySort	92,593	323	0.35
gallopLeft	88,332	511	0.58
gallopRight	78,034	476	0.61
mergeForceCollapse	58,567	475	0.81
ensureCapacity	50,707	321	0.63
pushRun	42,142	129	0.31
countRunAndMakeAscending	38,087	**0**	–
ensureCapacity-PredAbstr	37,943	343	0.90
invAccessible	15,202	38	0.25
TimSort	11,812	**0**	–
rangeReverse	7304	**0**	–
mergeAt	6330	794	12.54
minRunLength	2096	**0**	–
sort(c)	259	**0**	–
rangeCheck	225	**0**	–
**Average**	155,131	1533	(0.99)
**Median**	42,142	323	(0.77)

The average and median for the percentage of interactive rule applications is computed from the total average and median of (interactive) rule applications

Our experience with TimSort (and some smaller examples) is that the application of “if-then-else”-based state merging is most suitable when (1) there is a reasonably large remaining program left for execution after the merge, and (2) the merged states do not differ too much. If these criteria are not met, it can happen that the overhead introduced by the more complex expressions arising from the merging cancels the advantages. In this case, the proof size might increase (as in the case of mergeForceCollapse and ensureCapacity) and the resulting proof nodes might be harder to understand by human users. We therefore recommend to use state merging as early and locally as possible. Predicate abstraction is generally very likely to reduce the proof size; however, inferring suitable abstraction predicates is not an easy task.

### User Interaction

While KeY was able to handle six out of 19 methods of the TimSort class fully automatically, the remaining methods required user interaction. Table 3 shows the relation between the number of user interactions and the total number of rule applications in those proofs. On average, 0.99% of all rule applications were performed manually.

**Fig. 3:**
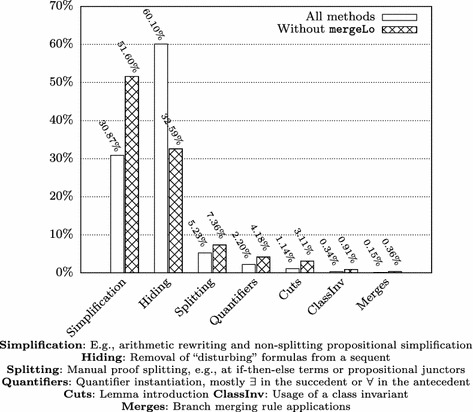
Proportions of interactive rule applications (grouped by type) for all TimSort methods and excluding mergeLo

Clearly, not all interactions were really necessary for finishing the respective proof obligations. Figure 3 illustrates the proportions of the types of manually applied calculus rules, once for all TimSort methods and once excluding the method mergeLo. (We discuss the particularities of this method in the subsequent paragraphs.) The categories of cut and quantifier-related rules sum up to roughly 3% (7% without mergeLo); 90% (84%) of user interactions reside in the categories “Hiding” and “Simplification”. Hiding refers to the removal of formulas in sequents that (could) distract the automatic strategies; simplification covers, for instance, arithmetic rewriting rules and non-splitting propositional simplification. That not all interactions are strictly necessary is partially caused by the fact that users tend to hide and simplify more formulas than absolutely necessary after the automatic strategies timed out on a proof branch.

Relevant input for investigating the effect of different “proving styles” of KeY proof engineers can be obtained from the data for the methods mergeLo and mergeHi. Those methods were, due to explosion of symbolic execution branches, unfeasible without state merging, and thus not proven in [13]. Listing 16 contains an excerpt of the code for both methods. It illustrates their complexity with nested loops, label breaks, and several method calls, but also their similarity. The code is entirely symmetric and performs the same operations for merging two adjacent “runs”; merely for optimization purposes, mergeLo is supposed to be called when the first run is smaller than the second one, while mergeHi is called otherwise.
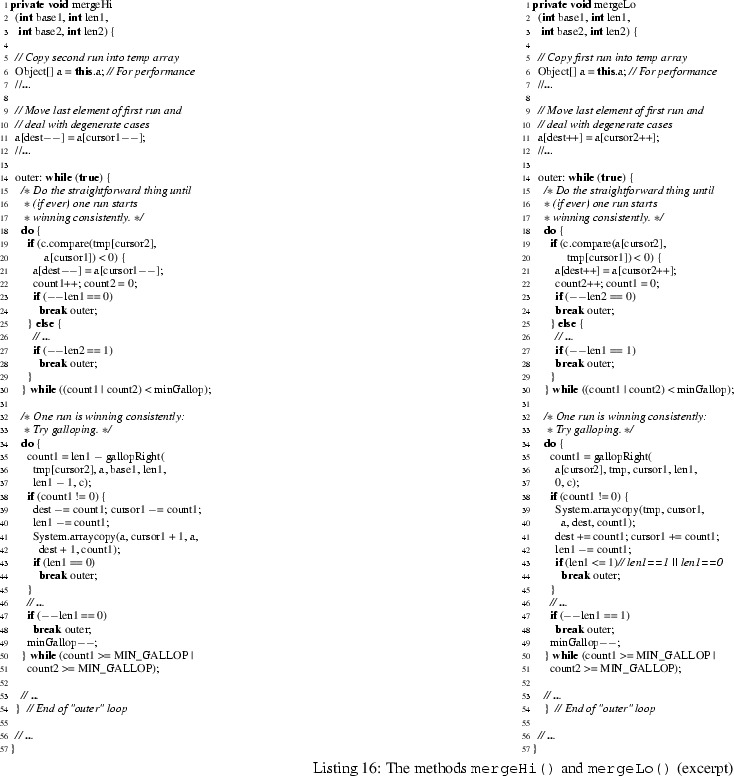

**Fig. 4:**
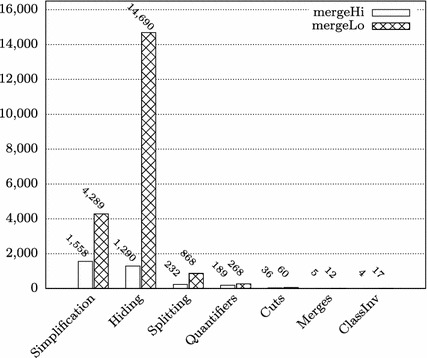
Number of interactive rules in mergeHi/mergeLo (grouped by type)

**Fig. 5:**
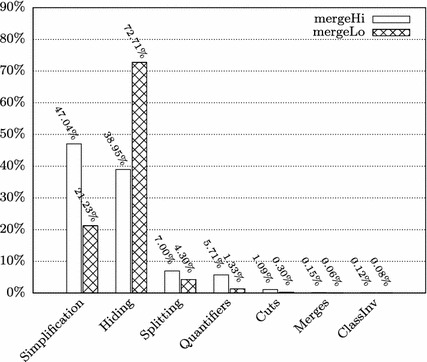
Distribution of interactive rules in mergeHi/mergeLo (grouped by type)

It would be reasonable to assume that the proof sizes and effort for both methods roughly coincide; however, the proof for mergeLo is more than three times as large as the one for mergeHi. The proofs were done by different teams, following different strategies. In the case of mergeHi, the strategy was to do a careful preparation of proof sequents by using hiding, simplification and useful cuts and quantifier instantiations early; whenever the strategies went into a disadvantageous direction, the proof was pruned back to keep it small. In the case of mergeLo, the automated search strategies were used more extensively. The consequence, however, was the necessity of even *more* simplification and hiding steps in the end. The effect of these different interaction strategies is visualized in Figs. 4 and 5 which depict the absolute and relative number of interaction types for both methods. Through the normalization by the total number of interactive rule applications, Fig. 5 provides some insights which do not immediately emerge from Fig. 4; for instance, it emphasizes the higher effort spent on “complicated” rules such as cuts, splitting and quantifier instantiation in mergeHi, while in the case of mergeLo, hiding is significantly more prominent. The latter can be explained by the chosen approach of relying more on the automatic strategies, which then inferred much more “disturbing” numeric equations etc.

Such considerations permit the conclusion that the shape of proofs may, especially for very complex proof obligations, depend much on the experience of the proof engineer. In either case, however, KeY ’s built-in search strategies usually perform at least 99% of all rule applications automatically.

A further manifestation of different interaction strategies, and the efficiency of the proof engineer can be observed in Table 4. It compares the proof statistics of mergeCollapse for the Android fix (method mergeCollapse_android) and our fix (method newMergeCollapse). The Android version was proven by the same team as mergeHi, while the new Java version (newMergeCollapse) was proven by the mergeLo team. The shorter proof for Android’s fix is despite the fact that the Android version involves a slightly more complex specification. (All other proofs did not need to be changed and are thus identical.)

In terms of person-months, the specification and verification for all methods excluding mergeLo and mergeHi took around three person-months. This was the proof effort for the analysis reported in [13]. A large part of this effort focused on iterating specifying and proving, until the right specifications were found. Afterwards, the new branch merging technique was implemented in KeY, which made another attempt at mergeLo and mergeHi feasible. Due to backwards compatibility issues (see Sect. 7 for details) the original proofs for the other methods had to be proven again in this new version of KeY. This had the benefit that it allowed us to analyze the effect of the branch merging technique. We exploited the branch merging technique extensively in the new proofs (see above). In terms of person-months, reproving all methods with the branch merging technique (i.e., excluding mergeLo and mergeHi) took around a week, compared to three person-months originally. The two main reasons were that no “specifying and proving” iterations were needed anymore (the specifications could just be reused from the original effort), and the use of the branch merging technique, which significantly reduced the proof effort.

For mergeLo and mergeHi, as reported above, they were proven by different teams using different strategies. The effort for mergeHi was around 2.5 person-days in total (this includes the proof as well as the specification). For mergeLo, the estimated effort is about 30 days in total, i.e., 12 times as much. The verification of those methods was mostly done independently of each other, while the specifications are as symmetric as the actual code. This emphasizes the impact of the level of experience and the decisions made by human users on the performance of semi-automatic program verification when it comes to very complex programs. To perform the task of a verification engineer requires substantial training, even if systems like KeY handle a lot of the work automatically.

**Table 4:** Verification effort for Android’s fix of method mergeCollapse()

Method	Rule apps	Interactive steps	Interactive steps (%)
mergeCollapse_android (Android version)	122,710	825	0.007
newMergeCollapse (our version)	348,774	1849	0.53

## Lessons Learned for Verification

We describe the problems encountered during the case study and how we overcame them. We outline how these experiences motivated to improve the KeY system. Some of these improvements had been added already in [13], some others only since then.

### Incremental Development of Specifications

Our aim was to formally verify library code used in the real world, so the analysed code was not written with *design for verification* [23] in mind. As a consequence, it contains many performance optimizations that tend to make verification harder: loop break-outs, redundant non-modular code that causes some highly complex control flow, as well as integer operations relying on Java’s integer overflow semantics.

Another obstacle was that only informal specifications were available, either in the form of natural language inside source code comments or in the algorithm description found in timsort.txt.^13^ These texts were sufficient to understand the ideas behind the algorithm and to comprehend the source code. The intended (but, as it turned out, insufficient) invariant was even explicitly mentioned. However, the descriptions were incomplete for a formal proof.

From this starting point, a formal specification in JML was developed in an incremental manner. After formalizing the natural language description and adding additional constraints, we started to verify the method contracts. When we failed, we improved the specifications based on the feedback obtained from KeY. For instance, KeY can produce symbolic counter examples. To illustrate this, consider the following unclosable goal, generated by KeY during an attempt to verify the original mergeCollapse implementation:
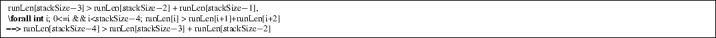


The quantified formula says: the element invariant holds except for the last five runs. The formula in the first line establishes the invariant for the final three runs. Nevertheless, the invariant is broken by the fourth but last run, as suggested by the fact that KeY can not prove the formula on the right-hand side of the implication. This information pinpoints where the invariant breaks (as analyzed in Sect. 3) and suggests how to fix the algorithm (as done in Sect. 4): add a test for index stackSize-4 “somewhere”. Thanks to symbolic execution, KeY produces proof trees that reflect closely the control flow of the program. This allows one to identify also *where* to add the extra check.

After we had realized that the intended class invariant was not preserved and did not hold, we scaled back our ambition to specify and verify full functional correctness of TimSort. We abandoned the goal to verify that the resulting array is indeed *sorted* and a *permutation* of the original, as well as the *stability* of the sorting algorithm. Instead we focused on the simpler verification task that no uncaught exceptions are thrown, including ArrayIndexOutOfBoundsException. Hence, the proposed fix actually eliminates the bug. Even so, specification and verification remained a rather complex and non-trivial task, as detailed in Sect. 5.

### The Best Choice of Integer Semantics

We mentioned above that certain parts of the TimSort implementation rely on Java integer overflows which are notoriously hard to reason about [8].

KeY supports three different kinds of integer semantics [1] that can be selected for a given proof. The general path from an integer arithmetic Java expression to its logic representation consists of unfolding complex expressions into a sequence of simpler expressions/statements until an elementary Java expression like i + j remains, which is then translated into the logic term $\mathtt {javaAddInt(i,j)}$. The axiomatisation of the function $\mathtt {javaAddInt}$ depends then on the chosen integer semantics:

1. Treat Java integral types as mathematical integers. With this semantics, the term $\mathtt {javaAddInt(i,j)}$ is rewritten into $\mathtt {add(i,j)}$, where the function $\mathtt {add}$ is axiomatized as the arithmetic addition on the integers. This semantics is neither correct nor complete, hence it is only used for teaching or academic purposes.
2. Permit successful verification of Java code only when either no overflow can occur, or when the value of overflowing integer operations does not influence the verification result. In our example, this semantics rewrites the term $\mathtt {javaAddInt(i,j)}$ into the conditional term
  $$\begin{aligned} \mathtt {if\ (inInt(add(i,j)))\ then\ add(i,j)\ else\ javaAddIntOverFlow(i,j)}\,, \end{aligned}$$
  whose conditional checks if the sum of $\mathtt {i}$ and $\mathtt {j}$ using normal arithmetic addition is within the range of Java’s int-type. If yes, the result of the normal addition is used, otherwise an integer typed term using the unspecified function symbol $\mathtt {javaAddIntOverFlow}$ is returned. The latter means that the result is some integer but we don’t know which one. This means a property can usually only be shown if the arithmetic operation does not overflow or if the result of the addition does not influence the validity of the property. This semantics is correct for the Java language specification, but not complete. It ensures that a Java program has the verified properties, but there are correct Java programs (relying on overflow) that cannot be proven.
3. A faithful model of the Java integer semantics with overflow and modulo operations. In this case the term $\mathtt {javaAddInt(i,j)}$ is rewritten into $\mathtt {addJint(i,j)}$ which is axiomatised as the Java addition with overflow. This semantics is correct and complete.

The first kind of semantics is unsuitable for our goal of verifying a real world Java library, because a proof that uses it would have little practical relevance. We decided to alternate between the second and third integer semantics, depending on the method under verification. For those methods that rely on integer overflows (ensureCapacity, gallopLeft, gallopRight), we had to use the (relatively cumbersome) third variant. We also chose this integer semantics for methods that use bitwise operators (binarySort, mergeHi, mergeLo, minRunLength and TimSort). For those methods, proving the absence of overflows (as required if we had chosen the second semantics), was more cumbersome than using the Java integer semantics. For all other methods, an overflow should not happen (and otherwise would be a bug) and we used the second semantics. Using that, one can easily avoid costly integer modulo operations (which cause excessive branching of the proofs) by simple range checks on the result.

For methods where the Java integer semantics had to be used, verification became tedious as modulo operations were then created for almost all integer expressions of that method, even for those where an overflow could not happen. We managed to simplify the verification task considerably by using lemmas that avoid the introduction of modulo operations in benign cases. These lemmas permit to create non-modulo operations in case the result of an operation is within the range of its Java integral type, or allow the easy removal of a modulo operator which is applied to an expression already known to be within the integral’s type range and thus being harmless, i.e., a lemma that expresses that if $a+b$ is known to be within a range *R* then $(a+b) \mod R$ is equal to $a+b$.

During the case study, the new lemmas were applied manually (or we relied on KeY ’s quantifier elimination strategies). In hindsight, we added the lemmas to KeY ’s rule base and tuned the proof search strategies to apply them automatically in an efficient manner.

### Proof Size Explosion

The most important lessons learned originate from the methods mergeLo and mergeHi (see Listing 16) whose proofs were elusive in [13]. They have over 100 lines of code each and exhibit complex control flow with nested loops, six breaks, and several if-statements. This leads to a memory overflow during proof attempts due to an explosion in the number of symbolic execution paths. For the present paper we investigated the reasons of this proof size explosion in greater detail. This triggered two improvements: a new symbolic execution rule for do-while loops, and a rule that allows merging of proof nodes.

*An Improved Rule for*do-while*Loops*.

The methods mergeLo and mergeHi consist mainly of one outer loop which includes two inner loops. The bodies of the inner loops contain several (nested) branching statements and break statements that redirect control flow. The inner loops are do-while loops, i.e., their body is executed at least once. Instead of providing specific loop invariant rules for each of Java’s loop statements (for, enhanced for, do-while and while), KeY provides program transformation rules that translate any loop into a while loop.

**Fig. 6:**
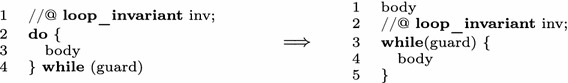
Schema of KeY ’s do-while transformation rule

The standard transformation rule for do-while loops, sketched in Fig. 6, copies the loop body in front of a while loop.^14^ It turns out that this transformation has major disadvantages, caused by the duplication of the loop body. It essentially requires proving the loop body *twice* (line 1 and line 4). Furthermore, if the copied loop body causes branching, the new while loop (lines 3–5) must be proven separately in each branch.

**Fig. 7:**
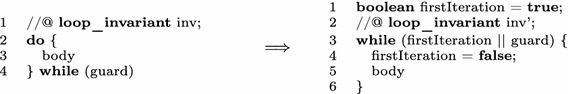
New and improved do-while transformation (simplified)

To avoid this redundancy, we implemented an alternative transformation rule that avoids duplication of the loop body. A Boolean variable firstIteration is declared that is initially set to true. To ensure that the loop is executed at least once, the guard is modified to the expression firstIteration || guard. The use of the short-circuit disjunction operator prevents the guard from being evaluated (with possible side-effects) in the first loop iteration. After entering the loop for the first time, the first statement in its body assigns firstIteration the value false. The new transformation rule is shown in Fig. 7. It has one disadvantage compared to the old transformation: the loop invariant must be able to use the newly introduced ghost variable firstIteration which is not known a priori. This can be done by entering the loop invariant manually when applying the rule. In addition, we added (after the case study) to our variant of the specification language JML the keyword $\backslash \texttt {firstIt}$ that can be used in do-while loop specifications to distinguish the first iteration from other iterations. The keyword is then replaced in the transformed invariant by the auxiliary variable firstIteration.

*A Uniform Framework for Symbolic State Merging* In previous work [13], we used *block contracts* to reduce the number of branches following branching statements like if. Block contracts are a generalization of method contracts which facilitate the annotation of arbitrary blocks of Java code with pre- and post-conditions. When executing a block based on its contract, one has to prove that the contents of the block satisfy the contract. Then, symbolic execution proceeds with the knowledge provided by the contract, abstracting away from the concrete behavior of the annotated block. However, this technique has two downsides: First, the applicability of using block contracts for mitigating state explosion is limited. Loops, for instance, can be exited at different break statements, leaving open several execution paths that begin at the same point in the program. This behavior is exhibited frequently in TimSort, for instance, in the methods mergeHi and mergeLo (see Listing 16). Second, block contracts are subject to the problems arising during incremental specification (Sect. 6.1): if the contract is invalid, this may become clear only after executing the content of the block and trying to verify the contract. Further, if the contract is too weak, the user might not notice this before finishing the execution of the remaining program after the block, and trying to verify the method’s post condition. In the case of large blocks or remaining programs, this constitutes a significant effort, and may require tedious backtracking and refinement of the block contract if there were problems.

To address the path explosion problem in a fundamental manner, we developed a uniform branch merging framework [22] that is highly flexible and can be applied on all nodes in a proof that point to the same statement in the program. The framework supports different state merging techniques of which some are abstraction-based and require the user to choose a suitable abstract domain, while others can be applied automatically and maintain full precision. A classic example for the application of state merging are the nodes arising after the execution of an if statement: They differ in the variables that have been changed in the if or the else block, and both point to the remaining program after the if statement. The choice of the merging technique determines how the differing values of those nodes are combined when merging them together. Two popular techniques are fully precise “if-then-else” merging, where both the differing values are remembered exactly, and predicate abstraction, offering an arbitrary degree of abstraction based on the chosen predicates. State merging overcomes the discussed disadvantages of block contracts: When using the fully precise merging technique, there is no need to provide a possibly complex specification for the block. Instead, nodes arising after the execution of a block which share the same remaining program to execute can be brought together without any further input by the user. This also spares the user the efforts for incremental specification of blocks. Furthermore, state merging is more flexible than block contracts: It can be applied in a wider range of scenarios (cf. the example of loops above), while still providing the possibility to abstract away from uninteresting concrete behavior through the supported abstraction-based techniques. As shown in Sect. 5, state merging effectively decreases the size of complex proofs. It enabled the verification of mergeLo and mergeHi which was previously out of reach.

## Challenges and Future Work

Challenges that need to be addressed in future work are (1) realization of proof reuse techniques, (2) backwards compatibility of theories, (3) deeper integration with SMT solvers, (4) full functional correctness. Some of these issues are in parts specific to our approach and tool, but we believe that at least the first three affect other approaches too, even though the symptoms might differ.

*Proof Reuse* A recurring scenario is the interplay between specification and verification. The most common event is that a method cannot be verified and its source code or specification needs to be fixed or amended. In formal verification, like in software development, the cost of fixing a bug becomes higher the later it is found. If the verification attempt was highly interactive until the point where the problem was identified, then all of the interactive steps have to be performed again. In its current version KeY supports only proof replay, but not proof reuse. This means once a branch cannot be replayed anymore, all work in that branch from this point onward is lost. The verification work for repeated proof attempts is not visible in the statistics, as it only considers the proofs’ size once it has been completed, but none of the proofs was done in the first iteration.

Adding proof reuse techniques that allow one to partially recover from this situation would reduce the verification effort considerably. Proof reuse techniques have been investigated for instance in [21], and earlier versions of KeY came with support for proof reuse [5]. The approach in [21] requires knowledge about all calculus rules which works only for systems with a small rule base. The approach implemented earlier in KeY focused on being able to reuse proofs after a program change, a scenario which did not occur in this case study, since once the bug was discovered, we focused on the verification of the fixed version.

*Backwards Compatibility of Theories* This topic is closely related to proof reuse discussed in the previous paragraph. One problem encountered when redoing the proofs from [13] was that calculus rules for some theories had been changed to make reasoning about those theories more effective, but now caused failure to reload the old proofs. This poses the question of how to establish backwards compatibility. One option would be to introduce versioning of the theory rules and to limit strategies to a newer version, while the old rules stay around for reloading. With time, this would increase the rule base considerably. Another option could be to provide the possibility to backup all rules used in a proof as part of the saved proof file, thus ensuring that these will be available when the proof is reloaded. In our case, better stability could also be achieved by recording proof macros instead of saving the whole proof object. Proof macros describe proof construction in a similar manner to tactics in proof assistants such as Isabelle [18]. At the time of our case study, KeY ’s proof macro framework was still in its infancy, but has since then considerably gained support. Nevertheless, proof macros would not solve all relevant problems since their reliability depends on the proof search strategies.

*Deeper Integration with SMT Solvers* KeY can delegate proof goals to SMT solvers. If such a proof goal can be closed by an SMT solver, KeY closes the goal by referring to the used SMT solver. The proofs in our case study do not include any proof goals closed by SMT solvers. The problem is that the SMT output is used as-is and cannot be further inspected. So the correctness relies on the implementation of the translation of proof goals into the SMT format and there is no way to validate the outcome with KeY. To mitigate this one could consider to implement back translation and reconstruction of SMT proofs for KeY in a similar manner as it is done for interactive proof assistants [7].

To be able to use SMT solvers successfully, KeY first needs to simplify the proof goals considerably. In particular, heap simplifications have to be done beforehand. The reason for this is that KeY ’s SMT translation cannot translate all kinds of rules into the SMT Lib format and hence the heap theory passed on to the SMT solvers is not complete. In future work, the translation should be extended to be able to translate more of KeY ’s theory axiomatizations and rules (lemmas) into the SMTLib format. In addition, it might be necessary to optimize this generic translation, which directly translates each axiom/rule as-is to a more specific ones that adds theories like the one for heaps in a formalisation that is easier approachable by SMT solvers.

*Full Functional Correctness* Although our work initially aimed at proving sortedness and the permutation property, we postponed these efforts after running into the specification and verification problems that revealed the bug discussed in this paper. We have not yet resumed work on verifying these properties. In our proofs, we also omitted a case that can only occur if the passed comparator does not implement a correct ordering relation. This required to slightly change the code by commenting out a branch in which an exception would be thrown if a bad comparator was detected in the methods mergeLo and mergeHi. It does not affect the proof that the bug is fixed, since if this branch would have been taken, execution finishes by passing the exception to the caller of the sort method.

*Other Future Work* We plan to investigate the relation between design, performance and verification effort in a systematic manner. A major problem in the verification effort described in this paper was the complex control flow of the methods mergeLo and mergeHi. As a first step we will implement and verify a more modular version of TimSort with simpler control flow. In particular, we are interested how this will impact the verification effort as well as the performance in terms of run-time. In addition, we plan to investigate how to reduce the considerable specification overhead by abstraction and specification generation techniques.

## Recommendations to Improve TimSort

### Binary Sort Issue

The Java implementation of TimSort relies on binary insertion sort to create runs of minimal length (see Sect. 2). During our verification effort in KeY we discovered an issue with the implementation of binary insertion sort.

The assertion in line 2 of Listing 17 is part of the original implementation. Suppose that lo, hi and start are all equal to $2^{31}-1$, the maximum value of integers. The increment of start in line 4 results in an overflow, so that start becomes negative. Then the loop guard start< hi evaluates to true, so that the loop is entered, which results in an ArrayIndexOutOfBoundsException exception on line 7.
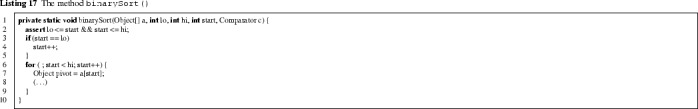


However, it does not lead to a bug, since in all places where the method is called in TimSort, it obeys a stronger precondition (the method is not exposed as public; it is only used internally by TimSort), hence the precondition can be sufficiently strengthened to rule out its occurrence during execution: we added lo< hi.

### Sorting of Array Segments

TimSort can be used to sort only a segment of the input array, by calling the method sort (Listing 1) with the desired bounds lo and hi. The sort method then calls the constructor of TimSort, which determines the length of runLen. However, this length is based on the length of the *entire* input array rather than the length of the segment that is to be sorted (see Listing 5, lines 5–7), which might be much shorter. This affects performance negatively. It could be repaired by adding a parameter int len to the constructor (and removing int len = a.length;), and instantiating this parameter to hi-lo in the call to the constructor in the sort method.

In contrast to the length of runLen, the minimal run length *is* based on the length hi-lo of the segment to be sorted, see lines 3 and 6 of Listing 1. However, as explained above, the computation of the length of runLen is based on the general minimal run length 16, which will never exceed the computed minimal run length in line 6. Hence, this discrepancy does not lead to further complications.

Such a modification would require to adapt the correctness proof accordingly which, unfortunately, includes a change of the class invariant. Indeed, the relation between the length of a and the length of runLen expressed in lines 3–6 of the class invariant (Listing 10) no longer holds.

There are at least two possible ways of adapting the current proof. First, one could add len as a ghost (instance) variable, and replace a.length by len in lines 3–6 of the class invariant. Second, one could remove lines 3–6 from the class invariant, add the relation between len and runLen to the invariant of the loop in the sort method, and finally add stackSizerunLen.length to the precondition of pushRun. Rather than redoing the proof from scratch, one would ideally obtain this new correctness proof by adapting the existing one according to one of the above two options. We leave this challenge as a case study for proof refactoring after a (minor) refactoring of the code.

## Related Work

Several industrial case studies have already been carried out in KeY verifying reference implementations of the Java Card API [16, 17] and the real-time Java API [2]. The implementation considered here and its proof is the most complex and one of the largest so far.

Polikarpova et al. [20] specified and verified the full functional correctness of the EiffelBase2 container library using AutoProof [25]. Their work is in parts complementary to ours and poses different challenges. To the best of our knowledge, their data structures, e.g., lists, sets and hash tables are much more elaborate than ours (we use only arrays), and main efforts were to relate these to proper mathematical abstractions and dealing with framing issues to make the specifications useful for clients using the libraries. However, the algorithms themselves did by far not reach the complexity of the TimSort implementation for Java discussed in this paper.

The first correctness proof of a sorting algorithm is due to Foley and Hoare [11], who formally verified Quicksort by hand. Since then, the development and application of (semi)-automated theorem provers has become standard in verification. The major sorting algorithms Insertion sort, Heapsort and Quicksort were proven correct by Filliâtre and Magaud [10] in Coq, while Sternagel [24] formalized a proof of Mergesort within the interactive theorem prover Isabelle/HOL.

De Gouw et al. [12] implemented counting and radix sort in Java and verified their implementation using KeY. They proved that the algorithms did actually sort the array and that the result is a permutation of the original array. This concerned self-written implementations, in contrast to the TimSort implementation discussed in this paper.

## Conclusion

Beyond the correctness result obtained in this paper, our case study allows us to draw a number of more general conclusions:

1. State-of-art formal verification systems allow us to prove functional correctness of actual implementations of complex algorithms that satisfy a minimum degree of structure and modularity.
2. Even core library methods of mainstream programming languages contain subtle bugs that can go undetected for years. Extensive testing was not able to exhibit the bug. Sect. 3 indicates why: the smallest counterexample is an array of 67+ million elements (with non-primitive type), and in [13] we show that it exhibits a very complex structure.
3. Software verification is often considered too expensive. However, precise formal specification allowed us to discover that the invariant is not preserved—in an afternoon. Sect. 6.1 shows that this fact also inevitably arises during a verification attempt with KeY. The combination of interactivity with powerful automated strategies was essential to formally verify the fixed version.
4. Static analysis and model checking are not precise, expressive and modular enough to fully capture the functionality of the involved methods. Expressive contracts are crucial to break down the problem into feasible chunks.

We conclude that functional deductive verification of core libraries of mainstream programming languages with expressive, semi-automatic verification tools is feasible. Further, it is clearly worthwhile: the OpenJDK implementation of sort() is used daily in billions of program runs, often in safety- or security-critical scenarios.

The infamous Intel Pentium bug cost a lot of revenue and reputation, even though, just as the TimSort bug, the actual occurrence of a defect due to it was extremely unlikely. Since then, formal verification of microprocessors became standard (e.g., [3]). Isn’t it time that we begin to apply the same care to core software components?

## Footnotes

1 Note that the main loop also contains a local variable runLen, which is used to keep the length of the currently processed segment.

2 http://bugs.java.com/view_bug.do?bug_id=8011944.

3 The increase to 24 had not been propagated to Android when [13] was written. Thus breaking the invariant and triggering the bug in Android was possible for relatively small arrays of size 65536.

4 See https://bugs.openjdk.java.net/browse/JDK-8072909.

5 See http://bugs.python.org/issue23515.

6 See comment at line 409 at https://android.googlesource.com/platform/libcore/+/android-6.0.1_r77/luni/src/main/java/java/util/TimSort.java.

7 See https://issues.apache.org/jira/browse/LUCENE-6293.

8 See https://github.com/timjb/haskell-timsort/commit/d9c3e1e454c7d338320f8c2aa12e882921d42828.

9 The JML expression $\backslash$old(e) refers to the value of the expression e at the time of entry into a method.

10 We chose our class invariant in such a way that all instance methods preserve it. However, it is also customary to allow private methods to temporarily break the invariant, as opposed to public methods. JML provides additional flexibility by means of the helper keyword, which allows one to mark those methods that need not preserve the class invariant.

11 The parameters runLen and runBase of pushRun shadow instance variables with the same name. In JML, to refer to the instance variables in specifications one prefixes this, just like in Java.

12 The proofs are available at www.key-project.org/timsort/.

13 http://svn.python.org/projects/python/trunk/Objects/listsort.txt.

14 The actual rule is somewhat more complex, as it has to deal correctly with breaks, continues, returns, exceptions and local variable declarations.
